# Added Sugar and Oral Health: A Position Paper of the Brazilian Academy of Dentistry

**DOI:** 10.3389/froh.2022.869112

**Published:** 2022-04-06

**Authors:** Carlos Alberto Feldens, Liana L. Pinheiro, Jaime A. Cury, Flávia Mendonça, Mario Groisman, Rafael A. H. Costa, Henrique C. Pereira, Alexandre R. Vieira

**Affiliations:** ^1^Department of Pediatric Dentistry, Universidade Luterana do Brasil, Canoas, Brazil; ^2^Private Practice, Rio de Janeiro, Brazil; ^3^Piracicaba Dental School, Universidade de Campinas, Piracicaba, Brazil; ^4^National School of Public Health, Fundação Oswaldo Cruz, Rio de Janeiro, Brazil; ^5^Federal University of Rio de Janeiro, Rio de Janeiro, Brazil; ^6^School of Dental Medicine, University of Pittsburgh, Pittsburgh, PA, United States

**Keywords:** dental caries, sugars, diet, policy, oral health

## Abstract

Excessive sugar consumption is the main cause of dental caries. Dental caries is highly prevalent and negatively impacts the quality of life at all stages. Furthermore, sugar consumption is associated with other noncommunicable conditions and diseases, such as obesity, diabetes, and cardiovascular diseases. The aim of this paper is to propose recommendations at the individual and population levels for health professionals, families, educators, stakeholders, and public officials to reduce the burden of dental caries and other noncommunicable diseases that are caused by the excessive sugar intake. A systematic search was performed in PubMed and Cochrane databases to investigate the effectiveness of strategies and policies aiming to reduce sugar consumption as well as the impact of different patterns of sugar consumption on the occurrence of dental caries. Reference list of the identified papers and practice guidelines were manually reviewed as well. Based on the best evidence available, the Brazilian Academy of Dentistry recommends not to offer sugars to children younger than 2 years of age, and to limit total sugar consumption to <25 g per day after 2 years of age. Furthermore, families should be informed to limit sugar exposure, sugar-free areas should be available, content of food labels and advertisement should be regulated, taxation of products with sugar should be introduced, and reformulation of foods and drinks to reduce concentrations of sugars should be considered.

## Introduction

Untreated dental caries in the permanent dentition is the most prevalent condition among all human diseases, affecting more than 2.5 billion people [1, 2]. A regional evaluation of dental caries showed marked social disparity, with the poor and disenfranchised significantly more affected [3]. These disparities are established very early in life, prior to and at the time children start elementary school [4, 5]. In the primary dentition, untreated dental caries is the single most common chronic childhood disease, affecting 621 million people worldwide, which corresponds to more than 50% of children under 6 years of age in the majority of countries, reaching nearing 100% in some countries [6].

Studies published in the last two decades showed the negative impact of dental caries in all aspects of life related to oral health in the infancy, school age, adolescence, adulthood, and among the elderly. Dental caries causes pain, impairs function, and has an impact in the emotional and social wellbeing [7, 8]. Dental caries affects all functions that comprise the current definition of oral health—the ability to speak, smile, smell, taste, swallow, and express emotions with confidence, without pain, discomfort or disease [9]. Further, dental caries impacts academic performance, leads to loss of working hours, and direct and indirect costs for the individual and society, including loss of productivity [2]. Untreated dental caries can also potentially lead to more dramatic outcomes, such as documented instances of blindness [10] and death [11].

Due to its complexity, dental caries requires different strategies to promote health and reduce the burden of the disease in the population. Dental caries occurrence depends on educational interventions, community fluoridation policies, and individual factors such as socioeconomic status, behavior, and biological factors [12]. The literature consistently shows that diet, with excessive sugar consumption, particularly sucrose (the sugar originated from sugar cane or beets), is the main cause of dental caries in all ages [13]. Hence, sugar continues to be highly consumed worldwide from the first year of life. The Foreign Agricultural Service of the US Department of Agriculture states that Brazil has been, for decades, the world's largest sugar producer and exporter, exerting major influence on global sugar supply and prices. Between May 2020 and May 2021, Brazilian sugar production achieved 42 million tons, of which 24.1% was destined to domestic consumption, which means an average sugar consumption of ~47.6Kg/inhabitant/year or 130.4g/inhabitant/day at the period [14].

In Brazil, foods and drinks high in sugar are consumed by almost all children at the age of 6 months, with long-term implications for general and oral health [15–17]. In this context, individual and collective strategies to reduce sugar consumption and the burden of non-communicable diseases have been tested in the last decade in different countries. Some promising results point to the need to gather this knowledge in order to propose policies to be implemented according to the context of each country.

Since dental caries is a sugar intake-based disease, the aim of this report is to propose recommendations at the individual and population levels, to promote action with the goal of reducing the burden of dental caries and other noncommunicable diseases that are caused by the excessive sugar intake.

## Methods

A PICO question (Population, Intervention, Comparator/s, Outcomes) strategy was carried out in PubMed and Cochrane to identify intervention studies and systematic reviews of intervention studies on the effectiveness of educational strategies to reduce sugar consumption (Intervention), compared to a control group (Comparator/s) in any age (Population) in the occurrence of dental caries (Outcome) [18]. In addition, a search on results (impact) of programs and implementation of protocols to reduce sugar consumption was done. Finally, another strategy using the PEO question (Population, Exposure, Outcome) to identify longitudinal studies, randomized clinical trials, and systematic reviews that ascertained in any age, the impact of different patterns of sugar consumption (exposure) on the occurrence of dental caries (outcome) [18]. A manual search on the reference list of the identified papers and practice guidelines was also done.

### Sugar as a Risk Factor to Dental Caries

Sugars as risk factor for dental caries may be described by different terminology depending on the context (scientific literature, protocols and recommendations, labels and regulations). The lack of consistency on how to describe sugars, even in the scientific literature, makes the understanding of the issue more difficult. Table 1 lists terms and definitions found in the literature.

**Table 1:** Terms and definitions used for sugars [19–21].

**Term**	**Definition**
Sugar	A sweet, crystalline substance, C12H22O11, obtained chiefly from the juice of the sugarcane and the sugar beet.
Total sugars	The term is used conventionally to describe the monosaccharides glucose, galactose, and fructose, as well as the disaccharides sucrose, lactose, maltose, and trehalose. Total sugars include all sugars in a food or beverage from any source, including those naturally occurring (such as fructose in fruit and lactose in milk, starch in vegetables) and those added to foods.
Naturally occurring sugars	Include those that are an innate component of foods (e.g., fructose in fruits and vegetables and lactose in milk and other dairy products).
Added sugars	Include all sugars used as ingredients in processed and prepared foods and sugars eaten separately or added to foods at the table. Sucrose and high-fructose corn syrups are the most commonly added sugars.
Intrinsic sugars	Sugars that are present within the cell walls of plants (e.g., naturally occurring sugars) and are always accompanied by other nutrients.
Extrinsic sugars	Those sugars not located within the cellular structure of a food and are found in fruit juice, honey, and syrups and added to processed foods.
Free sugars	Include monosaccharides and disaccharides added to foods and beverages by the manufacturer, cook or consumer, and sugars naturally present in honey, syrups, fruit juices and fruit juice concentrates.

The World Health Organization (WHO) usually uses and recommends “free sugars,” since noncommunicable diseases associate with this contextual meaning of sugars [19]. On the other hand, the American Heart Association uses the terminology “added sugars” to refer to the risk factor for cardiovascular diseases. Many studies investigated the effect of drinks with added sugars, since these drinks are an important portion of individual energy intake. Calories from drinks with added sugars have low nutritional content and high energy, giving a similar satiation perception than solid foods. As a result, energy consumption may increase leading to excessive weight gain [19].

The term “early sugar consumption” refers to the intake of added sugars by a child very early in life. The hazardous potential to health of diets with added sugars for infants may be bigger, since these diets are not preconized for this age group. Natural carbohydrates, such as the lactose found in milk and starch found in fruits and vegetables, are the best energy source to complement the diet of a child. There is some variation in the literature of the meaning of early diet, which can mean the first months of life (usually the first 6 months), the first year of life, or the first 2 years of life.

Added sugars, particularly sucrose, appears to be the best terminology in regards to oral health, since it directly relates to the establishment and progression of dental caries lesions. Some data, however, are still unclear, such as the reference to fructose as the added sugar in several products for human consumption in the United States [20]. We, in this paper, are using “sugar” with the meaning of added sucrose to foods and beverages. In practice, it is impossible to avoid that different nutrients are represented and distinct individual behaviors are present in different epidemiological studies, due to the limitation of questionnaires and interviews used, which capture a behavior that can constantly and quickly change.

### Dental Caries and the Etiological Role of Sugars From the Diet

Two aspects related to sugar consumption make dental caries worse and should be the focus of future designed interventions: early life sugar exposure and the high frequency of sugars in all ages. There is evidence of sugar intake during the first year of life and early childhood caries [22–24]. On the other hand, sugar consumption right after the eruption of the first teeth facilitates the establishment of a cariogenic microbiota, which is a predictor for future early childhood caries experience [25, 26]. The early exposure of a child to sucrose influences the child's preference for sweets, leading the child to favor foods and drinks with added sugars instead of healthier foods [27], which in turn contributes to the dental caries experience in the future.

Investigations with different populations over the decades have shown that diet rich in sugars have a role in the occurrence and severity of dental caries. The readily available sugars allows for the repetitive production of acids from the bacterial metabolism keeping the pH low and leading to the imbalance between the demineralization and remineralization of the enamel surface. The demineralization will overcome remineralization, leading to the formation and progression of dental caries, and this is further worsened by the continuous exposure to sugars from the diet [28, 29]. Data show a dose-response relationship between sugar consumption and dental caries; the higher the sugar intake, higher the caries experience and severity of the disease [30, 31].

The association between high dietary sugar intake and dental caries is independent from socioeconomic and other demographic factors. This association is the basis for recommending intervals between meals. Although unclear what can be considered safe, it is reasonable to assume that sugar consumption once or twice a day (desserts) is not associated with an important increase in risk of dental caries [32].

### Sugar Consumption: Common Risk Factor of Noncommunicable Diseases

Noncommunicable diseases are the main causes of death, and in many instances, these occur prior to 70 years of age [33, 34]. There is an association between sugar consumption and diabetes, cardiovascular diseases, nonalcoholic fatty liver disease, and cancer [13, 21, 33]. Due to these data, the Brazilian Ministry of Health, the American Heart Association (AHA), the European Society for Pediatric Gastroenterology Hepatology and Nutrition (ESPGHAN) and the International Association of Pediatric Dentistry recommend that children should not eat foods and drinks with added sugars (sucrose, fructose, and glucose) before the age of two [20, 21, 35, 36].

The World Health Organization (WHO) emphasizes the role of free sugars (sugars added to food and drinks and naturally present in foods, such as fruit juices and honey) in the incidence of noncommunicable diseases. The WHO recommends limiting the consumption of free sugars to up to 10% of the total energy intake, suggesting that limiting this consumption to 5% of the total food consumption brings an additional benefit to children and adults alike [13, 33]. This corresponds to ~25 g (six tea spoons or 100 kilocalories) of sugar per day per individual with a healthy BMI body mass index) and has the support of the AHA and ESPGHAN [20, 21]. Although these recommendations are based on the amount of sugars and dental caries depends on the frequency of consumption, it is reasonable to assume that the reduction of sugar amounts will lead to the reduction of frequency of sugar consumption and vice-versa.

Since knowing that added sugar consumption is growing, it is already at levels much higher than the ones recommended, and is a risk factor for dental caries and other noncommunicable diseases, intervention for the reduction of sugar consumption is the responsibility of all health professionals, including the dentist, foundations promoting the wellbeing, opinion leaders, and public decision makers.

### Individual and Community Interventions

Articles retrieved through the literature search included randomized clinical trials, mostly focused on behavior change strategies, and before and after studies, which investigated the impact of different policies and programs to reduce sugar consumption in a country or community. Overall, five strategies have been proposed to reduce sugar consumption and to promote oral health of the individual and communities.

### Educational Strategies for Families

At the individual level, there is moderate evidence from randomized clinical trials showing the benefit of delaying sugar intake and reducing its consumption in the first years of life [15, 37]. These educational strategies reduced the occurrence of relevant outcomes in Brazil, such as respiratory disease and dental caries, but depend on the frequency and vigor of these educational interventions, and the link between the family and health centers of health professionals [37–39]. In Hong Kong, incorporating diet counseling through motivational interview into dental care for adolescents improved oral health behaviors and prevented new dental caries lesions [40].

Although educating mothers and families regarding healthy eating may reduce disease risks, broader approaches not depending on “behavior changes” may be more effective in reducing sugar consumption, and ultimately health disparities [41]. The experience in several countries include promising interventions for reducing obesity and the burden of noncommunicable diseases, including dental caries [42].

### Taxation of Sugary Drinks and Foods

Taxing products with added sugars has been tested and tried in more than 40 countries, with evidence of reduction in the consumption of these products [43]. This intervention potentially may contribute to the reduction of sugar consumption and improving the health of the population by (a) increasing retail prices and reducing sales and purchases of taxes drinks and foods; (b) raising public awareness on negative health effects of sugars; (c) encouraging no added costs industry responses, such as product reformulation; and (d) generating government revenue, which can be directed toward services that improve population health [42, 44, 45].

France increased taxes of sugary beverages in 2012, which resulted in immediate reduction of the consumption of these products. United Kingdom started increasing taxes according to the excess of concentration of sugar in drinks and foods [42]. With super-taxation of sugary drinks in Ireland, which increased prices to the consumer by 10% caused a reduction of 11% in the consumption of these products. In Mexico, price increases of 10% of sugary drinks in 2014 caused an immediate reduction of 6% in the consumption of these products and a 12% reduction after 1 year. This reduction was even more pronounced (17%) in poor communities [42]. The increase of taxation of sugary drinks in Thailand had similar effects, particularly among individuals with lower socioeconomic status [46]. Another before and after study in South Africa showed a large reduction in taxed beverage intake one year after the implementation of a sugar-content-based tax. Among taxed beverages, sugar intake decreased from 8.8 g/capita/day pre-tax to 19.8 post-tax [45]. In Brazil, the federal government implemented a tax on soft drinks in 2013, with a rate of 27% for juice drinks, nectars, and other sugar sweetened beverages. However, the federal government decreased the tax rate in 2016 and 2018, contradicting global trends [17].

These experiences showed that taxation requires engaging stakeholders, such as politicians, the public and private sectors, the media, and health professionals. These strategies should include a broader agenda, beyond the health sector, and engage partners that can influence public decision making and involve the media [47].

### Reformulation of Foods With High Sugar Content to Reduce Sugar Concentration

The reformulation of foods by reducing sugar content during production is an attractive idea since it may reduce sugar consumption without behavior change [42]. This approach would be ideal for food products marketed to infants, which typically have sugar levels way above the recommended [48]. In the United Kingdom, a call for reformulation of foods that contributed the most for sugar consumption by children was made by the government. Between 2016 and 2019, a significant reduction in sugar consumption could be seen, particularly due to the reformulation of yogurts by reducing the concentration of sugars [49]. In Brazil, the Ministry of Health and the food industry signed an agreement in 2018 to reduce the amount of sugar in products to diminish the population-wide sugar consumption by 144,000 tons by 2025. However, there are no legal means in the country to regulate the execution of these agreements and the promised results are not likely to be achieved [17].

### Regulation of Advertisement and Labeling of Foods With Sugars

Advertisement and sales of products with sugars, as well as their labeling, influence the consumer behavior of families and the dietary habits of children [42]. The WHO and Pan American Health Organization made a series of recommendations regarding advertisement and commercialization of foods and nonalcoholic drinks for children. In Chile, it was implemented in 2016 the “Law of Foods,” which regulated packages of foods and drinks, disallowing the use of cartoons, and the sales of sugary drinks in schools. Furthermore, language for food and drink label warnings was normalized to improve the ability of the consumer to differentiate less healthy from healthier foods and drinks. Those measures not only aim to contribute with the reduction of sugar, salt, and fat consumption, but also to motivate the industry to reformulate their products. The result was a reduction of 24% in the consumption of sugars in Chile [50]. Ecuador and Peru followed by adopting new rules regulating packages of foods.

The addition of health warnings in the labels of sugary drinks in Peru, Uruguay, Mexico, Israel, Chile and some states of the United States resulted in a reduction of the sales of sugary drinks and positive changes in behavior, such as a different perception of risks to diseases causes by high sugar consumption and a reduction in the intent of consuming such products [51]. Brazil recently decreed a new food labeling legislation to help consumers better understand the nutritional information on labels, including the presence of added sugars, hopefully allowing for more informed food choices [17].

### Promoting Sugar Free Areas

Promoting areas that are sugar free, particularly in and around schools and pre-schools, has been proposed. In Australia, it is public policy that schools are not allowed to sell foods or drinks with excessive amounts of sugars or salt. In Brazil, it is law since 2009 that food planning in schools should be done by trained nutritionists, who should emphasize the restriction of sugars and fat. In addition, regulations included prohibition of sales of sugar sweetened beverages in schools. However, there are differences in compliance with these rules across Brazil [17]. Hungary, through the HAPPY (*Hungarian Promoting Programme in the Young*) program, put emphasis on drinking water rather than soda associated with increased taxation of sugary beverages. HAPPY reduced the consumption of soda among Hungarian children [44]. The Chilean law of Food Labeling and Advertising, which caused significant reduction of sugar consumption, included banning the sale of foods and beverages containing added sugar in schools and nurseries [50].

Simply recognizing a risk factor is not enough to change risk behaviors associated with disease. For decades, it is known that smoking is associated with a four-fold increase in heart attacks ands stroke, 20-fold increase in lung cancer, and a ten-fold increase in emphysema and chronic obstructive pulmonary disease. However, a marked reduction in smoking, particularly in Brazil, happened after implementation of broader interventions, such as advertisement and labeling restrictions, and increase in taxation. Data from 500 million male smokers showed that the biggest impact in smoking occurred when taxation was of 100% over price value [52].

On the other hand, policies and programs to reduce sugar consumption must be integrated and take into consideration the reality in each country and community. This includes not only the specification of these measures (e.g., definitions of products to be taxed; type of tax to implement; uses of the tax revenue; which products should be banned in schools), but also the participation of the actors involved [17, 42]. In this sense, dental professionals and research organizations should disseminate the evidence, through advocacy and direct actions. The measures should be supported by civil society engagement, based on the recognition that high sugar consumption is a public issue that has severe impacts on society. Governments need to develop strategies to ensure that these interventions are implemented and monitored, which includes capturing the impacts of sugar reductions [42].

The present analysis has limitations. In particular, the recommendations are based on a few randomized clinical trials, restricted to behavior change strategies to reduce sugar consumption. Thus, the reported benefits of upstream policies are based on observational studies, without a control group. However, it is not plausible to randomize people or clusters to some of the measures, such as increasing taxes on sugary products or regulating advertising. Furthermore, the mandatory implementation of these policies by governments is very new and the results are still short-term, and the current available evidence in limited.

## Conclusion

Dental caries is prevalent, unevenly distributed, and has an impact in quality of life of individuals and societies. However, the disease is highly preventable, but requires interventions that can lead to reduction of its primary cause: the excessive consumption of added sugars to the diet. Such interventions have the potential to reduce dental caries and other noncommunicable diseases that are linked to sugar consumption, such as diabetes and cardiovascular diseases.

### Future Directions and Recommendations

Based on what was exposed above, The Brazilian Academy of Dentistry recommends that foods and drinks with added sugars are not offered to children before they are 2 years of age, and no more than 25 g per day should be consumed, preferably with or right after meals. To achieve this goal, the following actions are proposed:

(a) To implement educational family interventions targeting the individual and communities, preferably at primary health centers.
(b) To promote sugar-free environments, prioritizing schools, pre-schools and the work environment.
(c) To regulate the content of labels of foods and beverages with added sugars.
(d) To restrict advertisement of products with sugars.
(e) To increase taxation of foods and beverages with sugars.
(f) To reformulate foods and beverages in regards to sugar content, to reduce sugar concentrations.

Future investigations should measure the long-term impact of different programs and policies, in order to assess whether the expected results in reducing sugar consumption are sustained. Furthermore, policy evaluation should not be restricted to the assessment of reducing sugar consumption, and also include measures of impact on clinically relevant outcomes such as dental caries and other non-communicable diseases.

## Author Contributions

CF wrote the first draft of the manuscript in Portuguese and critically revised the final submitted version. LP, JC, FM, MG, RC, and HP critically revised the manuscript and approved the final version that was submitted. AV critically revised the original version of the manuscript in Portuguese and generated the version in english and finalized the manuscript. All authors contributed to the article and approved the submitted version.

## Conflict of Interest

The authors declare that the research was conducted in the absence of any commercial or financial relationships that could be construed as a potential conflict of interest.

## Publisher's Note

All claims expressed in this article are solely those of the authors and do not necessarily represent those of their affiliated organizations, or those of the publisher, the editors and the reviewers. Any product that may be evaluated in this article, or claim that may be made by its manufacturer, is not guaranteed or endorsed by the publisher.
